# Mammary microbiota of dairy ruminants: fact or fiction?

**DOI:** 10.1186/s13567-017-0429-2

**Published:** 2017-04-17

**Authors:** Pascal Rainard

**Affiliations:** 0000 0001 2182 6141 grid.12366.30 ISP, INRA, Université de Tours, UMR1282, 37380 Nouzilly, France

## Abstract

Explorations of how the complex microbial communities that inhabit different body sites might contribute to health and disease have prompted research on the ways the harmonious relationship between a host and its microbiota could be used to keep animals healthy in their production conditions. In particular, there is a growing interest in the bacterial signatures that can be found in the milk of healthy or mastitic dairy cows. The concept of sterility of the healthy mammary gland of dairy ruminants has been challenged by the results of studies using bacterial DNA-based methodology. The newly obtained data have led to the concept of the intramammary microbiota composed of a complex community of diverse bacteria. Accordingly, mammary gland infections are not mere infections by a bacterial pathogen, but the consequence of mammary dysbiosis. This article develops the logical implications of this paradigm shift and shows how this concept is incompatible with current knowledge concerning the innate and adaptive immune system of the mammary gland of dairy ruminants. It also highlights how the concept of mammary microbiota clashes with results of experimental infections induced under controlled conditions or large field experiments that demonstrated the efficacy of the current mastitis control measures.

## The notion of mammary microbiota challenges the concept of mammary gland sterility

There is currently a lively interest among biologists in the interactions between hosts and the microbiota associated with mucosal or cutaneous epithelia. The human body hosts complex microbial communities whose composition is determined primarily by body habitat [1]. It is now widely accepted that the harmonious relationship between the host and its microbiota contributes to health. In particular, the microbiota plays a fundamental role in the induction, training, and function of the host’s immune system [2]. Most studies have dealt with the skin or gut microbiota, but other body sites are now considered as harbouring their own microbiota, such as the oro-pharyngeal, urinary and genital tracts and even the lung [3]. Recently, the mammary gland (MG) has also been included among these sites.

The widely held belief that the lumen of the healthy MG is sterile has been challenged recently. Although the MG has been considered a sterile organ, application of molecular methods to the quantification and sequencing of bacterial DNA has yielded results suggesting that there are commensal microbial communities within the MG [4, 5]. These results challenge the notion that the healthy MG is sterile, and they have led to a paradigm shift prompting some researchers to consider mastitis, which used to be considered a host-pathogen interaction driven by host and bacterial determinants, as a manifestation of dysbiosis, i.e. an imbalance of the mammary microbiota [4–6]. This novel concept is the rationale behind research programs aimed at defining the intramammary microbiota and is arousing interest in the impact of commensal microorganisms on the immune response to mastitis-causing bacteria, or intentional intramammary instillation of probiotics to cure or prevent mastitis.

There are many significant implications of the existence of an intramammary microbiota. However, they have not been clearly stated, analysed in depth, or discussed. The aim of this paper is to develop the implications of a mammary microbiota and to consider this paradigm shift in relation to the current views of the pathogenesis of infectious mastitis and of MG immunobiology, and to the current mastitis control measures.

It has long been considered that the MG is naturally free of resident bacteria and that the milk of a healthy MG is germ-free. The theory that milk within the healthy udder is germ-free was advanced in the years 1874–1878 [7]. Soon after, the theory that the udder is inhabited by a “normal flora” consisting of bacteria always found in the environment of cattle was put forward, as reported by Plastridge [8]. This theory was abandoned following studies showing that milk from healthy glands was normally sterile, on the basis of conventional bacterial culturing applied to aseptically taken milk samples. The researchers who were advocates of MG sterility stressed that precautions for aseptic milk sampling were of the utmost importance to get reliable results, stressing that obtaining sterile milk samples in the cowshed or milking parlour is impractical, and that the most reliable method of determining whether the bacteria have an intramammary source is by teat wall puncture [9].

Recently, with the advent of culture-independent methods of microbial identification, the concept of a sterile intramammary milieu has been challenged anew and more and more studies report that the healthy MG accommodates rather large and varied bacterial populations. Not all researchers and practitioners were satisfied with the bacteriological procedures in use previously. They have stressed the shortcomings of the classical bacteriological analysis, in particular the high rate of no-growth samples, i.e. milk samples taken from an inflamed MG that did not yield cultivable bacteria [10]. It was suspected that routine techniques were not adapted to the growth of many mammary pathogens, and alternative methods were proposed. In particular, detection methods based on the amplification of bacterial DNA were used. These new techniques provided results that contradicted the cultural methods: bacteria of many genera were found in milk from healthy MG [4, 11]. These results were interpreted as resulting from the existence of a mammary microbiota that would inhabit every MG, healthy or not [12]. This new concept had been developed in the last decade by some groups of researchers working on mastitis in breast-feeding women [5, 13].

The breast milk microbiota concept called into question the accepted view of breast sterility. This view had been stated in a World Health Organisation report [14]. In this report, it was considered that it is common knowledge that bacteria are often found in milk from asymptomatic breasts, with a bacterial spectrum very similar to that found on skin, including staphylococci and streptococci. It is noteworthy that despite careful techniques for collection, only 50% of milk cultures may be sterile, while others show “normal” colony counts ranging from 0 to 2500 colonies/mL. These bacteria may be skin contaminants or bacteria shed from colonized milk ducts. It was accepted that aseptically taken milk samples could be loaded with a few bacteria (<10^3^/mL milk) during their passage through the nipple ducts [15]. Bacterial colonization of the infant and breast is a normal process that takes place soon after birth. Both the mother’s milk ducts and the infant’s nasopharynx are colonized by a variety of organisms, some of them potentially pathogenic, such as *Staphylococcus aureus* [16].

This view was regarded as outdated by some researchers, and a new school of thought has emerged in the last decade, based on the increasing use of “omics” approaches. Although aseptic collection of human milk is questioned, culture-dependent methods have found bacteria in milk assumed to be aseptically collected. This has led to the notion of the human milk microbiota: it is considered normal that human milk from healthy women contains 10^3^–10^4^ cfu of diverse bacteria per mL [13]. The most commonly isolated bacterial species from human milk include *Staphylococcus epidermidis*, *S. aureus*, *Streptococcus mitis*, *Streptococcus salivarius*, *Lactobacillus salivarius*, *Lactobacillus fermentum*, *Lactobacillus gasseri*, *Lactobacillus rhamnosus*, *Bifidobacterium breve* and *Bifidobacterium bifidum* [13]. It is noteworthy that many of these species are members of the normal flora of the skin and the oro-pharynx. The number of cultivable bacterial species that can be found within one individual ranges from 2 to 18 different species, but this number has been substantially increased by the use of culture-independent techniques, based on the amplification of the variable regions in genes coding for 16S ribosomal RNA (16S rDNA), associated or not with pyrosequencing of the gene [5, 17]. From the data establishing the existence of a milk sample microbiome, some authors assumed the existence of a commensal microflora of human milk and of a mammary microbiota [13].

The mechanism by which bacteria reach the mammary gland has been the subject of debate and speculation. The proposed view is that bacteria from the gut microbiota would reach the mammary gland by an endogenous route [18]. It is speculated that bacteria taken up from the gut lumen by leucocytes such as dendritic cells or macrophages would be carried to the mammary gland by phagocytes migrating to the mammary gland by the haematogenous route, then making their way to the mammary gland lumen to be finally shed in milk. It has been shown that translocation of bacteria from the gut lumen to milk in mononuclear leucocytes may occur in lactating mice for a short period after delivery [15]. This entero-mammary pathway has been described for lymphocytes primed in the gut or the associated lymph nodes. These cells would then migrate to other mucosal sites, including the mammary gland [19]. This possibility is discussed below.

The concept of human mammary gland microbiota was proposed to be applied to the bovine mammary gland [20], and this idea was taken up in later studies. By using metagenomics pyrosequencing of bacterial 16S rRNA genes, a wide variety of bacterial species were found in each of the milk samples of mastitic and healthy cows [4]. It was also found that the microbiota of milk specimens derived from healthy cows was different from the microbiota of the mastitic specimens. This finding and the fact that members of several bacterial genera were found in every sample obtained from healthy quarters led the authors to postulate the existence of a microbiota indigenous to the bovine mammary gland [12]. It is noteworthy that *S. aureus* and *Streptococcus uberis* DNA were found in milk samples from healthy quarters with low somatic cell counts, leading the authors to postulate that these bacterial species that are known to exist on the skin or in the intestinal tract of the cow are part of the normal microbiota of the mammary gland. As in cases of clinical mastitis caused by one of these pathogens, these bacteria dominated the milk sample microflora [4], the conclusion was drawn that these infections were the result of dysbiosis of the mammary gland microbiota (or dysbacteriosis) rather than a mere primary infection [12]. In another study, an average of 30 different bacterial genera were found in samples from mastitic or healthy bovine mammary quarters [11]. It is noteworthy that the amount of DNA in samples from healthy quarters was so low that a DNA amplification step was necessary before polymerase chain reaction (PCR) amplification and 16S rRNA gene analysis. It is important to bear in mind that these two amplification steps make the procedure exquisitely sensitive, and prone to detecting the slightest contamination of the sample, which would be difficult to avoid even in a surgical ward. Intriguingly, this study showed significant differences in the bacterial populations in milk samples from quarters showing signs of clinical mastitis in comparison to milk samples from healthy quarters.

## The milk sampling issue: the contamination controversy

By using usual culture-dependent techniques and non-selective culture media, bovine milk samples taken from healthy uninflamed glands with thorough aseptic precautions yield only very low numbers of colonies, so that 50 µL samples are usually devoid of cultivable bacteria (<20 cfu/mL). Under field conditions, it is easy to contaminate milk samples: “milk drawn by hand from a healthy gland through the teat duct will nearly always contain bacterial contaminants from the teat duct, teat lesions, udder skin or the hands of the sampler” [9]. As a consequence, the volume of milk used to seed plates of non-selective medium, usually aesculin blood agar, is restricted to 50 µL, and more often 10 µL. More than 137 species of microorganisms are able to cause bovine mastitis [21], but every mastitis case is generally considered to be caused by one primary pathogen, because usually only one bacterial species is identified in milk samples from diseased glands [22]. Nevertheless, simultaneous infections by two different pathogen species are not rare, and three pathogens may be found in a small proportion of cases. Generally, samples yielding three or more than three bacterial species are suspected to be contaminated. Indeed, caution should be exercised in the interpretation of bacteriological analysis results. Hand collection of milk almost inevitably contaminates the sample with bacteria that colonize the distal part of the teat canal and the skin of the teat apex, or that simply soil the teat apex. A rich diversity of bacterial species can be found on teat apices [23]. Even extreme care used to disinfect the teat apex is not a foolproof guarantee that the sample will not be contaminated, in particular under field conditions. A procedure less prone to contamination is the use of a disposable collection bag fitted with a blunt cannula [24]. After careful disinfection of the teat apex, the cannula is introduced through the teat canal and the milk is aspirated. Another advantage of this technique is that it enables the cultivation of anaerobic bacteria. By using this approach, it was found that milk from healthy quarters yielded negative cultures; that most samples from classical clinical mastitis yielded only one bacterial species; and that samples from quarters with summer mastitis-like signs yielded an average of 3.4 bacterial species [25]. Another way of getting cistern milk by bypassing the teat canal is the trans-parietal route, using a syringe and needle. A great number of precautions must also be taken under field conditions, such as shaving and thorough disinfection of the udder skin to avoid contaminating the samples. This has been shown for blood culture tests: if these precautions are not taken, it is difficult to get uncontaminated blood samples [25].

The issue of milk sample contamination is compounded by the volume of milk sample necessary to perform the analyses: for bacteriological cultivation, the standard volume is reduced to 10 µL. It is noteworthy that pyrosequencing uses approximately 100 times (1 mL milk) and qPCR 35 times the amount of milk compared to classical culture, and these techniques detect both dead and live microorganisms [4]. Consequently, very careful collection of milk samples is even more important for DNA-based methods than for conventional culture methods [26]. As noted above, many of the bacteria found by the DNA-based techniques are common inhabitants of the skin, the gut, or the mouth (breast milk samples). Also, many of the bacteria composing the milk microbiota of healthy MGs have the potential for causing mastitis. This is particularly the case with bacteria of the genera *Staphylococcus*, *Streptococcus*, *Pseudomonas*, *Corynebacterium*, and *Burkholderia* [4, 11]. Absence of inflammation in these MG is thus unexpected. Bacterial communities commonly found in raw cow milk comprise bacterial genera that were also found in studies on the milk microbiota, such as *Sphingomonas* and *Stenotrophomonas* [11, 27]. This is not surprising, as one likely origin of the raw milk microbiota is the microflora of the teat skin that contaminates the milk during milking.

Apart from contamination at collection, the finding of bacterial DNA in milk samples could result from the presence of dead bacteria or bacterial fragments. This bacterial DNA could be either free in milk, or translocated into the MG lumen by migrating leucocytes, as discussed above. Importantly, beyond the sample contamination controversy, it is necessary to take into account all the consequences and implications related to the existence of an intramammary microbiota, in terms of pathogenesis of infectious mastitis, immunobiology of the MG, and dairy cow husbandry, including good hygienic practices.

## Mammary microbiota and the innate immunity of the MG

The immune response that develops in reaction to pathogens is shaped by the host-bacteria interactions that prevail in the infected organ [28]. The existence of a microbiota is an important determinant of the organ-specific regulation of innate immunity. Knowing whether the MG is devoid of a resident microbiota, or whether the healthy MG accommodates harmless bacterial inhabitants is thus of high significance and consequence.

### Milk is a nutrient-rich medium that supports the growth of bacteria

Milk is a medium permissive to the growth of many bacterial species. The most prevalent mastitis-associated bacteria are able to multiply in vivo with a doubling time of 20–30 min during the first few hours following entry into the MG [29]. Another indication that milk has a limited antibacterial efficiency is that it is easy to induce intramammary infections by introducing small numbers of microorganisms through the teat canal. It has been shown that if even very few (1–10 cfu) streptococci [30], staphylococci [31] or *Escherichia coli* [32] gain access to the teat cistern of uninflamed glands, bacterial multiplication takes place and infection ensues. When the defenses of the MG are compromised, it is common for the infecting bacteria to reach concentrations above 10^6^ or 10^8^ cfu/mL in milk [25, 29]. Although review articles listing the defenses of the bovine mammary gland generally conclude that milk is well equipped with antibacterial substances [33–35], it is of common knowledge that freshly drawn milk spoils in a few hours if not refrigerated. Cheese making has been developed by dairymen to overcome this propensity of milk to let unwanted microorganisms proliferate. The addition of beneficial microbiota is used to overwhelm the undesirable bacteria. The mere fact that it is possible to grow a “positive” microflora in freshly drawn milk demonstrates that milk has a limited inhibitory potential. Also, raw milk aseptically drawn from healthy glands has been used in vitro as a growth medium to test the activity of antimicrobials against mastitis-causing bacteria, without detectable interference by a putative indigenous microbiota [36].

One implication of the above considerations is that once they are within the lumen of a lactating MG, many bacterial species are able to proliferate and reach high concentrations, unless a prompt immune reaction hampers their growth. This puts a very high pressure on the gate-keeper function of the teat canal. As way of compensation to this vulnerability, the MG has the capacity to detect microorganisms. This is the task of the innate immune system.

### The mammary gland is poised to sense and to react to MAMPs

In a sterile organ, the triggering of receptors of the innate immune system (the Pattern Recognition Receptors, PRR) by the Microbe-Associated Molecular Patterns (MAMPs) activates inflammatory cascades that generate a quick antibacterial reaction, but this does not apply to organs such as the gut or the skin, which are laden with commensals or symbionts and constantly confronted with MAMPs [28]. Normally, the constant stimulation of the colonized epithelium induces a tolerance to the local microbiota. For example, the gut is tolerant to bacterial lipopolysaccharide (LPS) so it does not react to relatively high amounts of this MAMP. This is not the case with the bovine mammary gland, which responds to low amounts of LPS by an influx of leucocytes into milk [37]. In fact, the MG is well equipped to detect bacteria and the components they release: infusion of MAMPs into the MG through the teat canal elicits strong inflammatory responses [38–40]. Mammary epithelial cells express Toll-like receptors (TLRs) at their apical face, and are equipped to detect bacterial intrusion and to trigger an inflammatory response [41, 42]. This reactivity is at variance with the existence of an intramammary microbiota, as epithelial cells exposed to bacterial communities are usually poorly responsive to MAMPs, possibly to avoid excess reactivity to the microbiota. This is because “TLRs bear little ability to distinguish between commensal and pathogenic microbes as such organisms generally bear far more structural similarities than differences between them” [43].

### There is no mucus shield to protect the mammary epithelium from a microbiota

The mammary gland is not a mucosal organ, and in particular its epithelium does not secrete a mucus [44]. Mucus secreted by specialized cells of the mucosal epithelia plays an important role in the epithelium/microbiota interactions, by limiting direct contact of the bacteria with the epithelium lining [45]. In mouse models, defects in the mucus layer, such as genetic ablation of the major mucin MUC2, allow increased contact of commensal bacteria with intestinal epithelial cells and lead to spontaneous colitis [46]. This observation suggests that, as there is no mucus layer to keep commensal bacteria at a distance from the mammary epithelium, an intramammary microbiota would induce mastitis. Another role of mucus is to concentrate antibodies of the secretory immunoglobulin A (sIgA) type and antimicrobial peptides in the mucus shield [45]. The very low concentration of sIgA (0.1–0.2 mg/mL) in milk of dairy ruminants is at variance with the existence of an intramammary microbiota, because microbiota generally stimulate multiple pathways to drive secretory IgA production by plasma cells located in the lamina propria [44, 47, 48]. This low sIgA concentration and the paucity of IgA-producing B lymphocytes in the healthy MG do not stem from an inherent incompetence of the MG, because it is possible to induce the production of IgA in the MG of ruminants by intramammary antigenic stimulation [49]. This strongly suggests that there is no permanent antigenic stimulation inside the mammary gland.

### Absence or rarity of isolated lymphoid follicles

**Figure 1:**
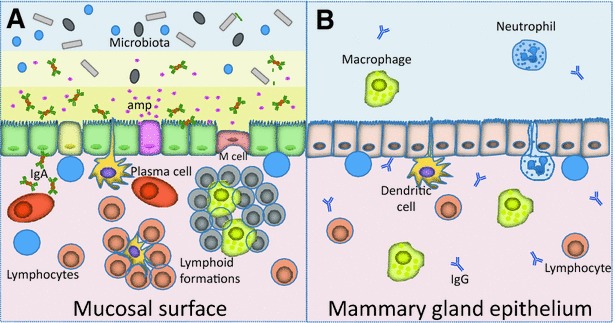
**The mammary gland epithelium is not a mucosal epithelium. A** Mucosal surfaces colonized by bacterial communities deploy distinct protective mechanisms. Within the simple columnar epithelium, goblet cells secrete mucus that covers the mucosal surface. The mucus inner and outer layers retain high concentrations of secretory IgA and host antimicrobial peptides (amp) secreted by epithelial cells or specialized cells such as Paneth cells. M cells transport luminal antigens to the dendritic cells beneath. Plasma cells secrete sIgA. T and B cells are present in the lamina propria, some are associated in mucosa-associated lymphoid formations. **B** The mammary gland epithelium is devoid of mucus, and bovine milk contains low-concentration of IgG. A few macrophages and neutrophils can be found in the lumen, but neutrophils are recruited en masse by inflammation when bacteria proliferate in milk. Bacterial intruders are detected by the epithelium comprised of epithelial cells and intraepithelial dendritic cells. Macrophages and T cells are present in the lamina propria, but organised lymphoid formations are absent from healthy glands.

Isolated lymphoid follicles (ILF) and associated T cell clusters (only visible microscopically) are common throughout the intestinal tract and occur in many mucosal locations. They are induced by exposure to antigens and microorganisms. ILFs in the upper respiratory tract are generally present only under conditions of antigenic challenge [50]. ILFs are also common at mucocutaneous transitions and near the ducts of secretory glands that empty onto mucosal surfaces [51]. ILFs have seldom been described in the bovine MG, mainly in the folds of the distal rosette of the teat cistern (Furstenberg’s rosette) [52]. ILFs were not detected in mammary tissue of MG that did not shed “conventional” mastitis-causing bacteria [53]. The rarity of ILFs in the MG constitutes another argument against the existence of an intramammary microbiota. Overall, there are major dissimilarities between a typical mucosal epithelium and the intramammary epithelium of dairy ruminants (Figure 1).

### Inefficiency of the entero-mammary pathway in ruminants

The entero-mammary pathway hypothesis states that some immune cells primed in the gut lamina propria can migrate to the mammary gland. This pathway has been shown to operate in rodents for lymphocytes and is also believed to operate in humans [19]. The entero-mammary pathway has been invoked to explain the transfer of gut lumen bacteria to the mammary gland [15]. Nevertheless, in cattle and sheep, a gut origin for MG lymphocytes is unlikely as mesenteric lymph node cells do not migrate to the MG [54]. It is unlikely that the gut-mammary pathway operates in dairy ruminants, because the MG of ruminants is not part of the common mucosal immune system as originally defined in monogastric species [44, 55]. Although the endogenous route may explain MG infection and the excretion of intracellular pathogens such as *Mycobacterium tuberculosis* or *Brucella* in the milk or milk cells of systemically infected animals, it is widely held that the teat canal forms the main portal of entry of microorganisms to the udder [8, 56]. It is noteworthy that bearing in mind the very low bacterial numbers necessary to induce intramammary infections, the continued ingress of viable bacteria-laden macrophages or dendritic cells from the gut or other sites into the MG lumen would pose a formidable threat to the MG.

Another origin of bacterial DNA in milk could be the passage of dead bacteria or circulating bacterial components from blood to milk. It is known that bacterial components can be found transiently in blood and that translocation of bacterial peptidoglycan to the bone marrow occurs [57, 58]. As the MG is richly vascularized and filters huge amounts of blood during lactation, it can be envisaged that some of these circulating bacterial components find their way into the milk. Yet, two considerations contradict this view. First, the mammary epithelium border is almost impermeable, preventing the crossing of even small molecules both ways (i.e. albumin from blood to milk, and lactose from milk to blood), although food proteins can be found in breast milk [59]. Second, the MG epithelium is very sensitive to MAMPs such as endotoxins, lipoproteins, peptidoglycan fragments, bacterial DNA or RNA. One possibility then is that the bacterial components, and among them 16S RNA, are shuttled in milk within phagocytic cells. These cells, monocytes or neutrophils, patrolling the mammary tissue, would occasionally traverse the epithelium and be shed in milk. Whole bacteria could even be carried by phagocytes, as long as live bacteria were not released in milk, in which case mastitis would ensue. Interestingly, according to the hypothesis of bacterial DNA as a cargo of migrating leucocytes, in the case of mastitis many blood leucocytes are recruited into milk, thus increasing the probability of recovering bacterial DNA in milk. This is in keeping with results showing the absence of bacterial DNA in the milk of non-infectious mastitis and the presence of DNA of many bacterial types in the milk of breast-infected women [60].

In conclusion, the existence of an intramammary microbiota seems incompatible with the established knowledge of the innate and adaptive immune system of the MG.

## Mammary microbiota compatibility with the five-point mastitis control plan

The question arises of the compatibility of the concept of intramammary microbiota with the mastitis control practices developed and endorsed by researchers at the National Institute for Research in Dairying (NIRD, UK) in the 1960s [61]. Specifically, is the mammary microbiota compatible with systematic dry cow therapy and teat dipping in disinfectant solutions? The routine use of disinfectant teat dipping after milking has been established as the best technique to reduce substantially new infections rates at the herd level [62]. Large field experiments indicated that the main direct effects of teat dipping were to reduce general skin contamination and prevent teat orifice infections [62, 63]. The main purpose of a teat dip is to destroy contaminating pathogens at the teat apex and the external opening of the canal, thus preventing infection of the teat canal. It ensures that any microbial community of the teat apex is compromised by the routine disinfectant teat dipping. Yet, this impact on the teat microbial colonisation results in a proven reduction in new intramammary infection rates [64]. Thus the widely accepted efficacy of post-milking teat dipping discredits the potential efficiently protective effect of a teat microbiota. Systematic or blanket drying-off therapy, i.e. intramammary treatment of all udder quarters with long-acting antibiotics at the beginning of the dry period, has been recommended for a long time because it proved to reduce efficiently the level of infection at the herd level [62, 65]. The products used are mainly effective against Gram positive bacteria, yet there is no increase in the prevalence of infections by Gram negative bacteria associated to their use [63, 65, 66]. Blanket drying-off therapy with long-acting (more than three weeks) antibiotic concentrations has the potential to interfere durably with any putative intramammary microbiota, and microbiota disruption by antibacterial products is known to favour dysbiosis, which may increase the susceptibility to infections after the cessation of treatment [67]. The proven efficacy of blanket dry cow therapy in reducing the incidence of new intramammary infections is another circumstantial evidence of the inefficiency of the putative intramammary microbiota, and is compatible with the idea that such a microbiota does not exist.

## An implication of mammary microbiota: use of probiotics for the mammary gland

According to the concept of mastitis as a manifestation of dysbiosis, i.e. an imbalance of the intramammary microbiota, the use of probiotics to re-equilibrate the microbiota appears as a possible corrective measure. Oral probiotics for the treatment of breast infections have been evaluated [68]. The oral route of administration is not likely to operate effectively in polygastric animals such as ruminants, especially since the entero-mammary pathway is poorly operative in these species. This is probably why probiotics for the bovine MG have been administered through the teat canal. Several *Lactobacillus* species or strains of *Lactococcus lactis* have been used as intramammary probiotics. The probiotic *L. lactis* DPC 3147 has been used as an alternative non-antibiotic treatment of mastitis, and its injection into the MG induces a sizeable inflammatory response [69]. In fact, *L. lactis*, although a Generally Regarded As Safe bacterium (GRAS), is a mastitis-causing pathogen [70, 71]. Intramammary inoculation of a commercial probiotic mixture of *Lactobacillus acidophilus* and *Lactobacillus casei* to cure mastitis has been found not to be efficient and caused a local inflammatory response [72]. Intramammary infusion of 10^6^ cfu of *Lactobacillus perolens* induced a mild inflammatory response, but its capacity to treat or prevent intramammary infections has not been demonstrated [73]. It appears that probiotic bacteria trigger an inflammatory response from the MG, and this is probably why they have been used for therapy rather than for prevention of intramammary infections (IMI). Concerning the prevention of new infections by pre-existing and long-lasting colonization of the MG by bacteria, an experiment of nature in relation to intramammary probiotics is the effect of the so-called minor pathogens *Corynebacterium bovis* and coagulase-negative staphylococci on the incidence of IMI by major pathogens such as *S. aureus*, streptococci and *E. coli*. Although quite a few studies reported some protective effect by minor pathogens, others have found the converse or no effect, and several ancient and recent reviews conclude that a protective effect would be of low magnitude in any case [74–77]. It is noteworthy that the efficiency of the alleged intramammary microbiota would be low, since a few (1–100) *S. aureus* or *E. coli* cfu are enough to cause mastitis with a success rate above 90% [32, 78, 79].

Probiotics are supposed to act by competitive exclusion. Certain milk bacterial strains have the capacity of inhibiting the growth of mastitis-causing bacteria in vitro, possibly through the production of bacteriocins, but this effect requires high concentrations of the interfering bacteria [80]. Yet the normal mammary microbiota is reported to comprise less than 3–4.7 log_10_ CFU/mL [6, 20]. The low concentration of milk bacteria in healthy glands and the frequent renewal of milk make the effective production of bacteriocins unlikely. Competitive exclusion has never been shown in milk in conditions mimicking the in vivo situation.

Two different logics conflict on the reactivity of the MG to bacteria. If we posit that an intramammary microbiota is a feature of the normal MG, then there must be a state of tolerance of the microorganisms composing the microbiota by the intramammary epithelium lining. Therefore, it could be possible to harness this microbiota or the tolerance to some microorganisms to enhance the resistance or resilience of the MG to mastitis-causing pathogens through the use of well-chosen probiotics. Although regulatory T cells have been found in the mouse mammary gland [81], published results to date do not support this possibility. Alternatively, if we posit that the normal MG is a sterile organ, then we can expect that any microorganism penetrating the lumen of the gland would trigger a response from the MG as soon as the threshold of reactivity was reached, because a sterile organ is not poised to tolerate a microbiota. Convergent data support this alternative view, casting doubt on the potential of probiotics as a potential preventive solution for mastitis control.

## The concept of mammary microbiota needs thorough examination

There is accumulating evidence that complex communities of microbes play a fundamental role in controlling many aspects of host physiology. A spate of studies reflects the excitement that pervades the scientific community, giving rise to the emergence of “microbiomics” accompanying the microbiota upheaval. To avoid being carried away by this hype, it is appropriate to apply a healthy dose of scepticism to microbiome science [82]. The novel concept of mammary microbiota has not previously been examined with regard to its implications in terms of mammary immunobiology. The proponents of the intramammary microbiota theory have not discussed the implications of their studies in relation with the current mastitis control practices and the ancient and most recent developments in mammary gland immunobiology. It appears that the intramammary microbiota concept is inconsistent with the properties of milk as a growth medium, the reactivity of the bovine MG to MAMPs, the absence of mucosal firewall to bacteria, and the paucity of organized lymphoid tissue in the lamina propria. Overall, the existence of an intramammary microbiota seems incompatible with the known data concerning the status of the innate and adaptive immune system of the MG. In addition, the mammary microbiota does not dovetail with data from experimentally induced mastitis and the success of the mastitis control measures currently in use. These discrepancies between the concept of mammary microbiota and the established knowledge of mammary infection immunobiology cast doubt on the efficacy and practicality of the intramammary probiotics approach to controlling mastitis. Although the existence and importance of a teat apex microbiota deserves attention, it is the opinion of the author that the existence of an intramammary microbiota is a fiction that could cause confusion and interfere with practices that have proved useful for mastitis control.
